# Functional coupling constrains craniofacial diversification in Lake Tanganyika cichlids

**DOI:** 10.1098/rsbl.2014.1053

**Published:** 2015-05

**Authors:** Masahito Tsuboi, Alejandro Gonzalez-Voyer, Niclas Kolm

**Affiliations:** 1 Evolutionary Biology Centre, Department of Ecology and Genetics/Animal Ecology, Uppsala University, Norbyvägen 18D, 75236 Uppsala, Sweden; 2 Laboratorio de Conducta Animal, Instituto de Ecología, Universidad Nacional Autónoma de México, Circuito Exterior S/N, Ciudad Universitaria, UNAM, D.F. 04510, México; 3 Department of Zoology/Ethology, Stockholm University, Svante Arrhenius väg 18B, 10691 Stockholm, Sweden

**Keywords:** functional coupling, constraints, phylogenetic comparative analysis, geometric morphometrics, rate of evolution

## Abstract

Functional coupling, where a single morphological trait performs multiple functions, is a universal feature of organismal design. Theory suggests that functional coupling may constrain the rate of phenotypic evolution, yet empirical tests of this hypothesis are rare. In fish, the evolutionary transition from guarding the eggs on a sandy/rocky substrate (i.e. substrate guarding) to mouthbrooding introduces a novel function to the craniofacial system and offers an ideal opportunity to test the functional coupling hypothesis. Using a combination of geometric morphometrics and a recently developed phylogenetic comparative method, we found that head morphology evolution was 43% faster in substrate guarding species than in mouthbrooding species. Furthermore, for species in which females were solely responsible for mouthbrooding the males had a higher rate of head morphology evolution than in those with bi-parental mouthbrooding. Our results support the hypothesis that adaptations resulting in functional coupling constrain phenotypic evolution.

## 1. Background

Functional coupling, the phenomenon where one structural system is required to perform multiple functions, is a universal feature of organismal design [1,2]. Such multi-functionality has been suggested to constrain the trajectory and rate of phenotypic evolution [3], whereas innovations that increase the evolutionary flexibility within a structural system can enhance the potential for diversification [4–6]. To date, however, the evolutionary impact of functional coupling on the rate of phenotypic diversification has rarely been investigated.

Mouthbrooding in teleost fishes has evolved from an ancestral state of substrate guarding, in which parents typically spawn and guard their eggs on a sand substrate or stone substrate or in rock holes/crevices [7]. The evolutionary transition to mouthbrooding offers an excellent opportunity to test the functional coupling hypothesis because mouthbrooding introduces a novel function to the cranium, which is originally adapted for feeding. Previous studies have found that uni-parental mouthbrooding is often accompanied by sexual dimorphism in craniofacial anatomy [8,9]. Additionally, a trade-off between reproduction and feeding was reported in cardinalfishes [10] and cichlids [11], suggesting that the functional coupling of feeding and brooding may impede morphological diversification [12]. Although these studies indicate that mouthbrooding has considerable influence on craniofacial diversification, studies that address the link between mouthbrooding and morphological diversification while considering the effect of shared ancestry are lacking.

The cichlid fishes of Lake Tanganyika are a textbook example of adaptive radiation [13] and display remarkable variation in body morphology [14] and brood care [15]. The eco-morphological diversity of Lake Tanganyika cichlids provides two separate contrasts that can be used to test the functional coupling hypothesis. The first contrast is between mouthbrooders and substrate guarders. In accordance with the functional coupling hypothesis [10], we predict that substrate guarding cichlids will present a faster rate of head shape evolution than mouthbrooding cichlids. The second contrast is within mouthbrooding cichlids, where males in species with bi-parental care perform both brooding and feeding, while males in species with maternal care do not brood the eggs or the offspring [15]. Therefore, we predict that males in species with maternal care will have a faster rate of head shape evolution than males in species with bi-parental care.

## 2. Material and methods

We used geometric morphometrics to quantify the head shape of 37 species of Lake Tanganyika cichlids. Details of morphological data acquisition are described in [16]. Briefly, we digitized nine homologous landmarks and seven semi-landmarks along the edge of the forehead. Subsequently, generalized procrustes analysis (GPA, [17]) was performed. GPA translates landmarks to the same origin, scales landmarks to the same centroid size and rotates landmarks around the centroid to minimize Euclidian distances among specimens to obtain size-standardized average shapes of each species. We first performed GPA for each species including both sexes. Subsequently, the same procedure was repeated using data for males (*n* = 22) and females (*n* = 26) of mouthbrooders to obtain morphological measurements for a comparison between bi-parental and maternal care species. Information on brooding ecology and sex of the parental care provider was obtained from the literature (electronic supplementary material, table S1).

All phylogenetic comparative analyses were performed using the R statistical environment [18]. Our phylogenetic tree was a subset of 500 trees drawn from a Bayesian phylogenetic reconstruction based on mitochondrial sequences downloaded from Genbank [19]. We employed stochastic character mapping [20] to visualize possible histories of character transition in brooding ecology and the sex of the care provider using the phytools package [21]. Using a transition matrix with unequal rates for ancestral state estimation, we sampled 100 character histories per tree. In order to test whether the rate of head shape evolution is different between groups of cichlids with distinct brooding strategies, we performed a simulation-based comparative analysis [22]. This analysis first employs phylogenetic transformation [23] and the transformed data are used to estimate a multivariate evolutionary rate parameter 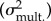 based on the Euclidean distance between each species and the origin of the phylogeny separately for the groups under study. Subsequently, the ratio of the rate parameter between groups is obtained 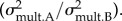 According to our hypotheses, the grouping was made based on either brooding strategy or sex of the care provider. The observed ratio is then tested against the simulated null distribution of the ratio of rate parameter under a uniform evolutionary rate model. The number of simulations to generate a null distribution of the ratio of the evolutionary rate parameter was set to 999. A *p*-value of 0.05 (i.e. more than 95% of the simulations show a lower ratio of the rate parameter than the observation) was employed as the cut-off point for statistical significance. The comparison of evolutionary rate for high-dimensional data was performed using the geomorph package [24].

## 3. Results

The stochastic character mapping revealed that a transition between mouthbrooding and substrate guarding occurred once (figure 1*a*), while transitions between maternal and bi-parental care occurred five times (figure 1*b*). We found that the rate of head shape evolution was significantly faster in substrate guarders (*n* = 9, 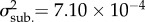) than in mouthbrooders (*n* = 28, 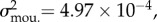 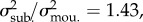 *p* = 0.003). Substrate guarders had more upward-pointing mouths with straight forehead outlines (i.e. between maxilla and anterior end of the dorsal fin) while mouthbrooders had more horizontally pointed mouths with curved forehead outlines (electronic supplementary material, figure S1). Within males of mouthbrooders, the rate of head shape evolution was also significantly faster in species with maternal care (*n* = 13, 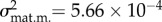) than in species with bi-parental care (*n* = 9, 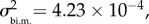 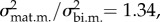 *p* = 0.03). Males of maternal brooders had upward-pointing mouths and dorsally positioned eyes, while males of bi-parental brooders had horizontally pointed mouths and ventrally positioned eyes (electronic supplementary material, figure S2). Finally, for females of mouthbrooders, the ratio of the evolutionary rate parameter between species with maternal care (*n* = 16, 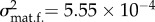) and bi-parental care (*n* = 10, 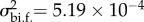) was not significantly different (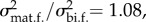 *p* = 0.52).

**Figure 1.**
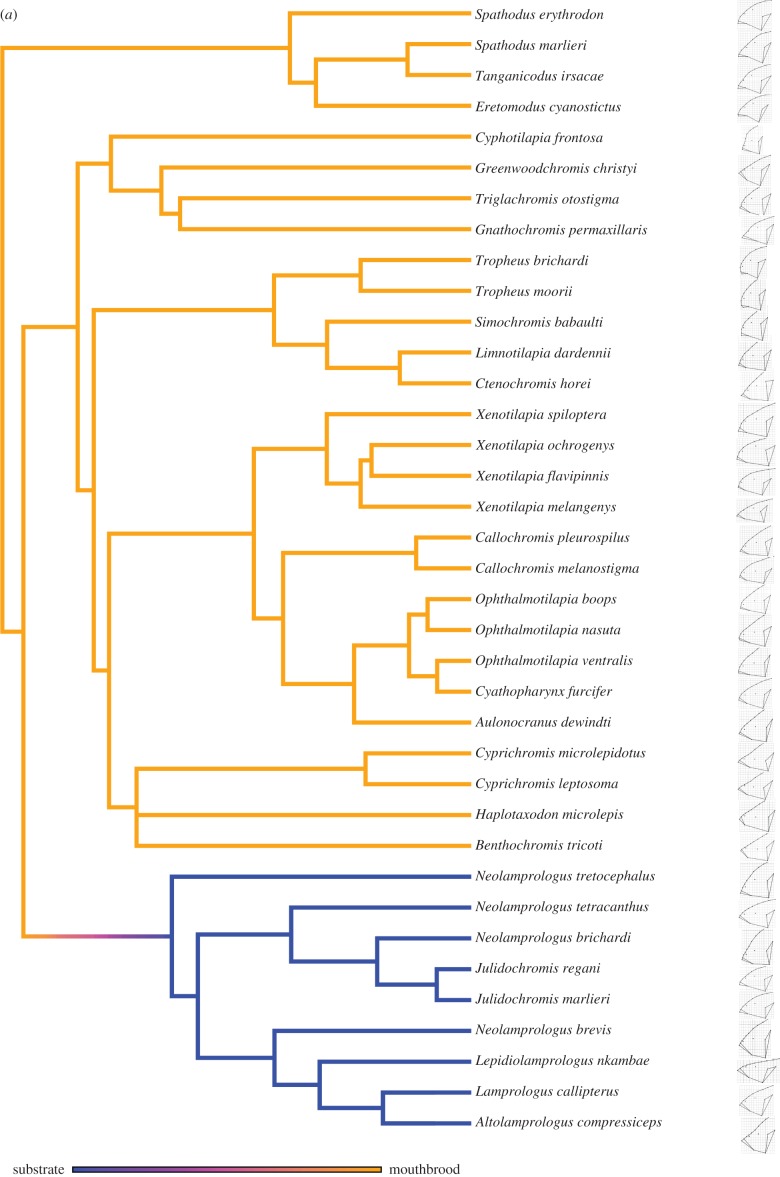
A molecular phylogeny of the Lake Tanganyika species used in our study with simulated character transitions in (*a*) the form of care (substrate guarding in blue and mouthbrooding in orange) and (*b*) the sex of the care provider (bi-parental care in yellow and maternal care in purple). A consensus configuration (i.e. an average shape) (*a*) for each species pooling both sexes and (*b*) for males (left) and females (right) is also provided.

**Figure 1.**
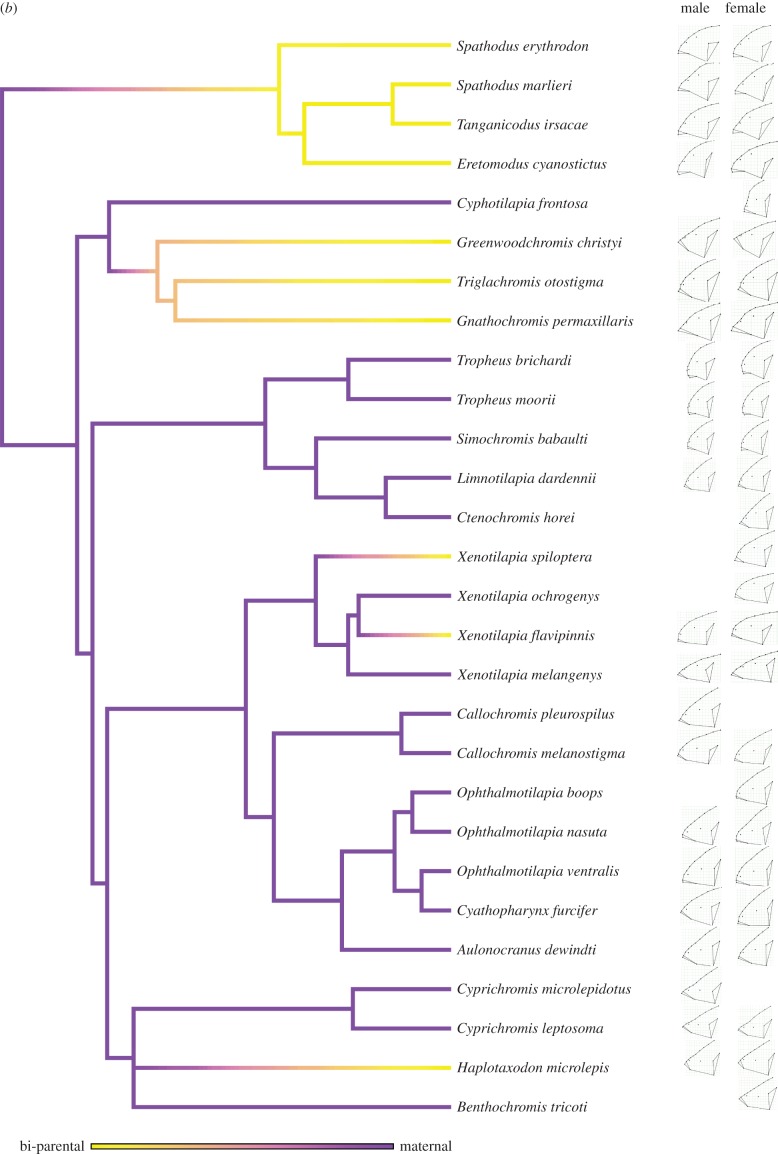
(*Continued*.)

## 4. Discussion

We demonstrate that mouthbrooding cichlids have a slower rate of head shape evolution than substrate guarding cichlids, in line with the functional coupling hypothesis [10,11]. Given that substrate guarding is the ancestral state in this lineage [7], this result suggests that the ecological transition to mouthbrooding involved sacrificing the evolutionary versatility of the craniofacial system. Our sex-specific analysis within mouthbrooders further reinforces this conclusion. We found that males of maternal care species have a faster rate of head shape evolution than males in bi-parental care species, while the difference was absent within females. Together, our results suggest that the multi-functionality associated with mouthbrooding constrains morphological diversification in Lake Tanganyika cichlids.

The key selection pressure in forming the tremendous variation in fish craniofacial diversity is trophic adaptation ([25,26], but see [27]). Given that the performance of both mouthbrooding and feeding is associated with head morphology [10,11], we speculate that mouthbrooding has a profound influence on trophic adaptation. Specifically, our result suggests that the decreased potential for morphological diversification in mouthbrooding fish might constrain trophic diversification. Furthermore, considering the critical importance of eco-morphological niche specialization during the adaptive radiation of African cichlids [28], our study indicates that mouthbrooding may constrain the rate of speciation. Future studies investigating the rate of ecological diversification and speciation in association with mouthbrooding will provide additional tests of the general implications of functional coupling on diversification patterns and processes.

## 5. Conclusion

Our phylogenetic comparative analyses provide support for the hypothesis that mouthbrooding operates as a constraint on craniofacial diversification [10,11]. More generally, our results suggest that functional coupling may play an important role in ecological diversification and speciation. The transition to mouthbrooding has occurred also in nine other families of fish [9]. Future investigation using these additional groups of mouthbrooding fishes and a variety of ecological transitions associated with novel functions in other vertebrate taxa will test the generality of the idea that functional coupling is an important constraint on vertebrate diversification.

## Supplementary Material

Additional analysis

## Data accessibility

The doi for our data is 10.5061/dryad.s1kg8.
