# Supplementary material for: Functional coupling constrains craniofacial diversification in Lake Tanganyika cichlids
Source: Biol Lett. 2015 May;11(5):20141053. doi: 10.1098/rsbl.2014.1053 (PMC4455731; doi:10.1098/rsbl.2014.1053)
Supplement: Additional analysis [file rsbl20141053supp1.pdf]

Functional coupling constrains craniofacial diversification in Lake Tanganyika cichlids

Masahito Tsuboi, Alejandro Gonzalez-Voyer and Niclas Kolm

Electronic Supplemental Material

Additional analysis

In order to investigate the pattern of shape variation associated with different brooding strategies, we performed GPA (please see main text for details) using morphoJ package version 1.06d [1] for six different groups of species separately; mouth brooder, substrate guarder, males of bi-parental mouth brooder, males of maternal mouth brooder, females of bi-parental mouth brooder, and females of maternal mouth brooder.

Shape differences between mouth brooders and substrate guarders, between males of bi-parental and maternal brooders, and between females of bi-parental and maternal brooders are given in figure S1, S2, and S3 respectively. Substrate guarders had more upward-pointing mouths with a straight forehead while mouth brooders had more horizontally pointed mouths with a curved forehead (figure S1). Head morphology of males of maternal brooders typically had upward-pointing mouths and dorsally positioned eyes while males of bi-parental brooders have horizontally pointed mouths and ventrally positioned eyes (figure S2). Finally, the difference in head morphology between females of maternal and bi-parental brooders was represented by the angle of mouth and the position of eyes (figure S3).

References

[1] Klingenberg, C.P. 2010 MorphoJ: an integrated software package for geometric morphometrics. *Molecular Ecology Resources* 11, 353-357. (doi:10.1111/j.1755-0998.2010.02924.x).

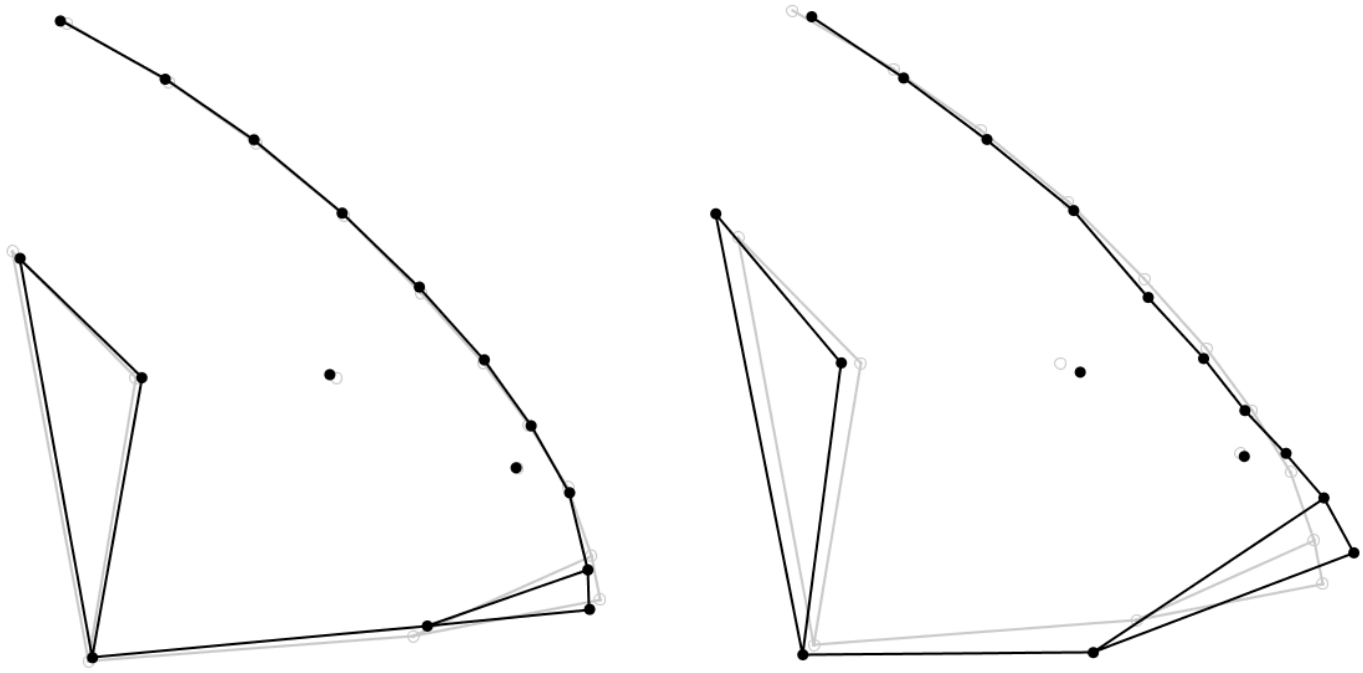

(a) Mouth brooder (b) Substrate guarder

Figure S1 Head shape difference between mouth brooder and substrate guarder. Average head shape of (a) mouth brooding cichlid species (n = 28) and (b) substrate guarding cichlid species (n = 9) in our study are given in solid line and points. A grey line and open pointes represent average shape for all species (n = 37) to visually guide the shape differences.

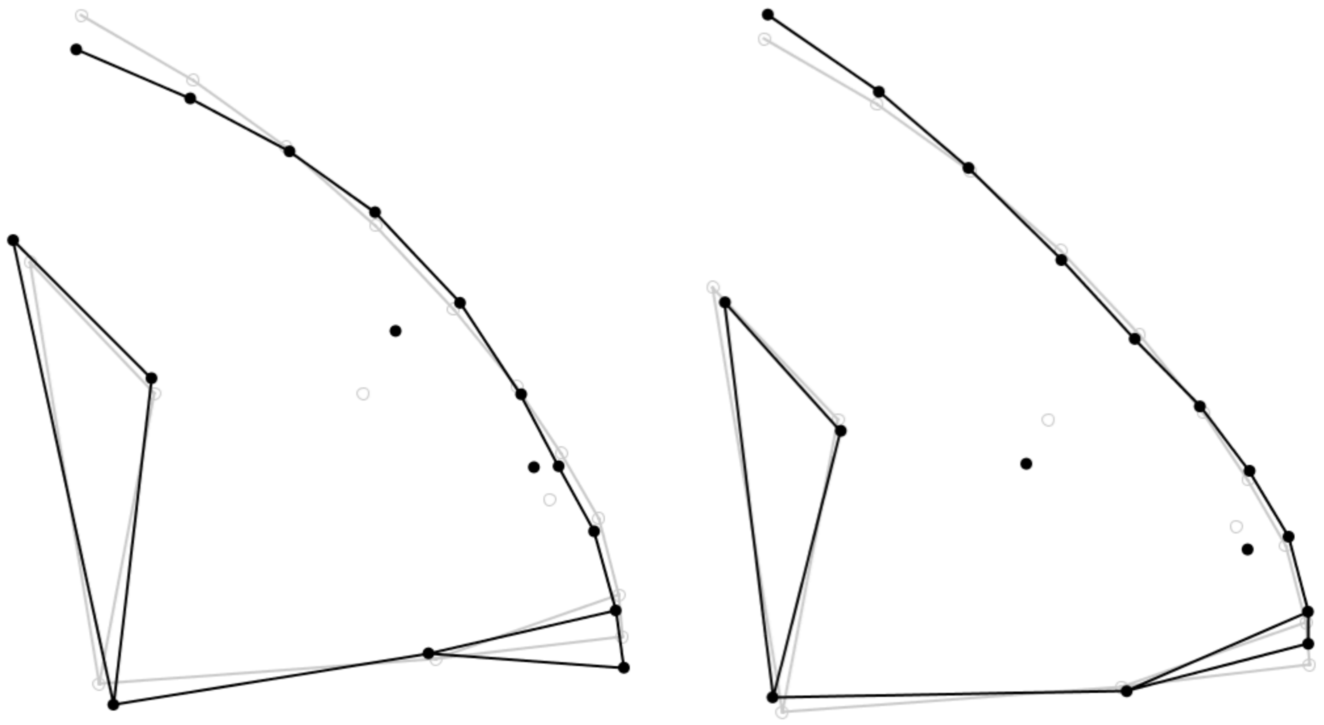

(a) Males of bi-parental brooder (b) Males of maternal brooder

Figure S2 Head shape difference between bi-parental brooder and maternal brooder within males. Average head shape of (a) males of bi-parental brooding cichlid species (n = 9) and (b) males of maternal brooding cichlid species (n = 13) in our study are given in solid line and points. A grey line and open pointes represent average shape for all species (n = 22) to visually guide the shape differences.

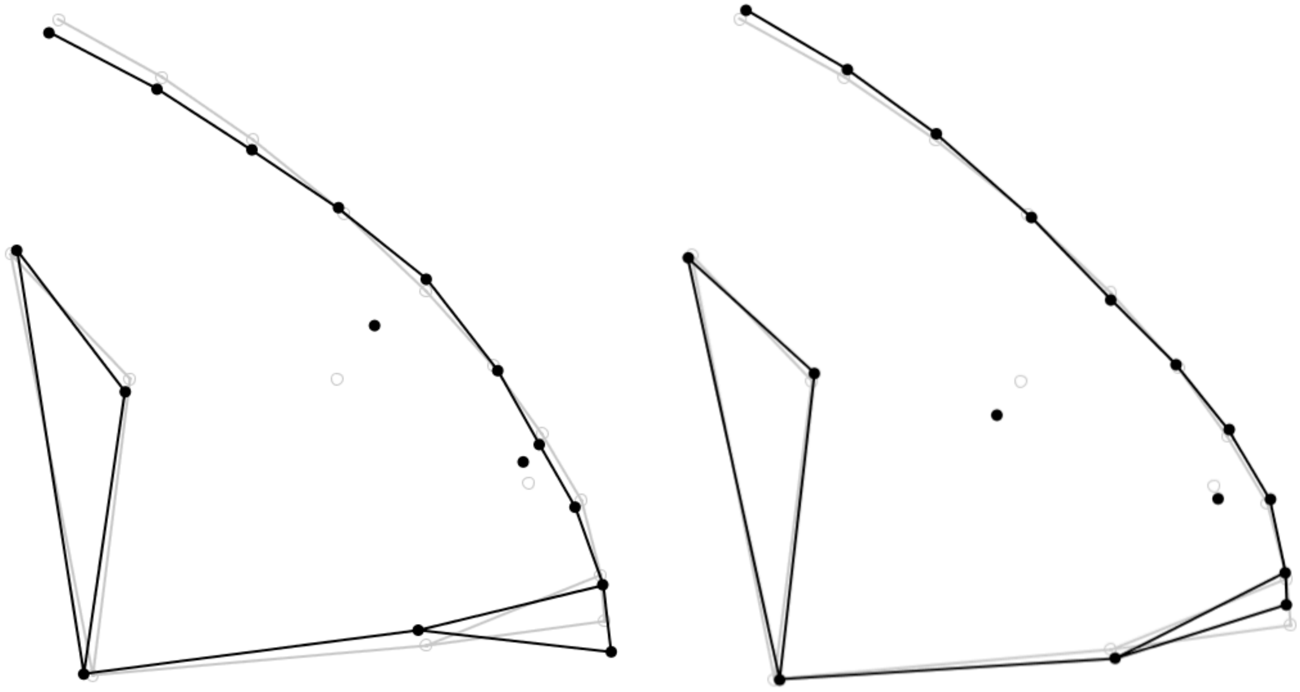

(a) Females of bi-parental brooder (b) Females of maternal brooder

Figure S3 Head shape difference between bi-parental brooder and maternal brooder within females. Average head shape of (a) females of bi-parental brooding cichlid species (n = 10) and (b) males of maternal brooding cichlid species (n = 16) in our study are given in solid line and points. A grey line and open pointes represent average shape for all species (n = 26) to visually guide the shape differences.

Table S1 Ecological information of the species used in our study. Species name, sample size, sex of care provider, form of care, and references are presented.

Species	Sample size		Sex of care provider	Form of care	Reference
	Male	Female			
Altolamprologus compressiceps	1	5	Maternal	Substrate guarding	Koning (2005)
Aulonocranus dewindti	2	2	Maternal	Mouth brooding	Koning (2005), Kuwamura (1986)
Benthochromis tricoti	0	2	Maternal	Mouth brooding	Koning (2005)
Cyphotilapia frontosa	0	1	Maternal	Mouth brooding	Koning (2005), Kuwamura (1986)
Cyathopharynx furcifer	1	2	Maternal	Mouth brooding	Koning (2005), Kuwamura (1986)
Ctenochromis horei	0	1	Maternal	Mouth brooding	Koning (2005), Kuwamura (1986)
Cyprichromis leptosoma	4	2	Maternal	Mouth brooding	Koning (2005), Kuwamura (1986)
Callochromis melanostigma	2	1	Maternal	Mouth brooding	Koning (2005)
Cyprichromis microlepidotus	5	0	Maternal	Mouth brooding	Koning (2005)
Callochromis pleurospilus	4	0	Maternal	Mouth brooding	Koning (2005)
Eretmodus cyanostictus	3	1	Bi-parental	Mouth brooding	Koning (2005), Kuwamura (1986)
Greenwoodchromis christyi	1	2	Bi-parental	Mouth brooding	Koning (2005)
Gnathochromis permaxillaris	2	1	Bi-parental	Mouth brooding	Koning (2005)
Haprotaxodon microlepis	4	2	Bi-parental	Mouth brooding	Koning (2005), Kuwamura (1986)
Julidochromis marlieri	1	5	Bi-parental	Substrate guarding	Koning (2005)
Julidochromis regani	2	4	Bi-parental	Substrate guarding	Koning (2005)
Lamprologus callipterus	3	0	Maternal	Substrate guarding	Koning (2005)
Limnochromis dardennii	4	2	Maternal	Mouth brooding	Koning (2005)
Lepidiolamprologus nkambae	5	1	Bi-parental	Substrate guarding	Koning (2005)
Neolamprologus brevis	2	1	Maternal	Substrate guarding	Koning (2005)
Neolamprologus brichardi	1	6	Bi-parental	Substrate guarding	Koning (2005)
Neolamprologus tetracanthus	1	0	Maternal	Substrate guarding	Koning (2005)
Neolamprologus tretocephalus	4	3	Bi-parental	Substrate guarding	Koning (2005)
Ophthalmotilapia boops	0	2	Maternal	Mouth brooding	Koning (2005)
Ophthalmotilapia nasuta	2	2	Maternal	Mouth brooding	Koning (2005), Kuwamura (1986)
Ophthalmotilapia ventralis	2	2	Maternal	Mouth brooding	Koning (2005), Kuwamura (1986)
Simochromis babaulti	2	3	Maternal	Mouth brooding	Koning (2005), Kuwamura (1986)
Spathodus erythrodon	3	1	Bi-parental	Mouth brooding	Koning (2005), Kuwamura (1986)
Spathodus marlieri	2	4	Bi-parental	Mouth brooding	Koning (2005), Kuwamura (1986)
Tropheus brichardi	1	3	Maternal	Mouth brooding	Koning (2005), Kuwamura (1986)
Tanganicodus irsacae	2	2	Bi-parental	Mouth brooding	Koning (2005), Kuwamura (1986)
Tropheus moorii	2	2	Maternal	Mouth brooding	Koning (2005), Kuwamura (1986)
Triglachromis otostigma	2	4	Bi-parental	Mouth brooding	Koning (2005), Kuwamura (1986)
Xenotilapia flavipinnis	3	1	Bi-parental	Mouth brooding	Koning (2005), Kuwamura (1986)
Xenotilapia melanogenys	3	1	Maternal	Mouth brooding	Koning (2005), Kuwamura (1986)
Xenotilapia ochrogenys	0	5	Maternal	Mouth brooding	Koning (2005), Kuwamura (1986)
Xenotilapia spiloptera	0	5	Bi-parental	Mouth brooding	Koning (2005), Kuwamura (1986)

References

Koning, A. 2005 *Back to Nature: Guide to Lake Tanganyika cichlids*. EL Paso, Cichlid Press

Kuwamura, T. 1986 Parental Care and Mating Systems of Cichlid Fishes in Lake Tanganyika: a Preliminary Field Survey. Journal of Ethology 4: 129-146
